# Real-world effectiveness of burosumab vs oral phosphate and active vitamin D in adults with X-linked hypophosphatemia

**DOI:** 10.1093/jbmr/zjaf063

**Published:** 2025-05-02

**Authors:** Pablo Florenzano, Erik A Imel, Aliya A Khan, Zhiyi Li, Marc Vincent, Takanobu Nomura, Stanley Krolczyk, Ben Johnson, Leanne Ward

**Affiliations:** Department of Endocrinology, School of Medicine, Pontificia Universidad Católica de Chile, Santiago 8320165, Chile; Departments of Medicine and Pediatrics, Endocrinology, Indiana University School of Medicine, Indianapolis, IN 46202, United States; Division of Endocrinology and Metabolism, McMaster University, Hamilton, ON L8S 4L8, Canada; Kyowa Kirin Inc, Princeton, NJ 08540, United States; Kyowa Kirin Inc, Princeton, NJ 08540, United States; Kyowa Kirin Co Ltd, Tokyo 100-0004, Japan; Ultragenyx Pharmaceutical Inc, Novato, CA 94949, United States; Kyowa Kirin International plc, Marlow SL7 1HZ, United Kingdom; Children's Hospital of Eastern Ontario, Department of Pediatrics, University of Ottawa, Ottawa, ON K1H 8L1, Canada

**Keywords:** disease and disorders of/related to bone, disorders of calcium/phosphate metabolism, therapeutics, osteomalacia and rickets, PTH/Vit D/FGF23

## Abstract

In X-linked hypophosphatemia (XLH), *PHEX* gene variants lead to elevated FGF23 production, resulting in hypophosphatemia, osteomalacia, osteomalacia-related fractures, osteoarthritis, enthesopathy, spinal stenosis, and symptoms of pain, stiffness, and decreased physical function. Burosumab is an anti-FGF23 monoclonal Ab approved for XLH treatment. Randomized studies comparing oral phosphate/active vitamin D (Pi/D) to burosumab in adults are lacking. This analysis, which utilized real-world data from the prospective, Americas-based XLH Disease Monitoring Program (NCT03651505), evaluated the effectiveness of burosumab vs Pi/D, based on changes from baseline to the year 1 visit in serum phosphate, 1,25(OH)_2_D, PTH, Western Ontario and McMaster Universities Osteoarthritis Index (WOMAC) scores, Patient-Reported Outcomes Measurement Information System Physical Function (PROMIS PF), and Timed Up and Go performance outcome. Two cohorts of adults with XLH who either began burosumab between baseline and the year 1 visit (*n* = 65) or were on Pi/D (*n* = 74) at study entry and did not receive burosumab were included. Inverse probability of treatment weighting was employed to adjust for potential confounding due to baseline cohort differences. At the year 1 visit, mean (SE) change from baseline was significant for burosumab vs Pi/D in serum phosphate (0.78 [0.08] vs 0.15 [0.14] mg/dL; *p* < .001), 1,25(OH)_2_D (19.41 [3.39] vs 5.49 [3.43] pg/mL; *p* = .011), PTH (−13.82 [5.00] vs 11.79 [8.10] pg/mL; *p* = .006), WOMAC pain (−7.50 [2.34] vs 4.47 [3.23]; *p* = .004), WOMAC physical function (−5.68 [1.96] vs 6.77 [4.85]; *p* = .006), and WOMAC total (−7.78 [2.06] vs 3.15 [3.37]; *p* = .005) scores, PROMIS PF (1.51 [0.73] vs −1.64 [1.11], *p* = .018), and TUG (−1.19 [0.42] vs 0.55 [0.43] s, *p* = .011). A trend towards improved WOMAC stiffness was observed for burosumab (−10.16 [2.85] vs −1.79 [3.68]; *p* = .086). In this real-world analysis of adults with XLH, burosumab treatment was associated with improved biochemical parameters, pain, physical function, and mobility compared with Pi/D.

## Graphical Abstract

**Graphical Abstract:**
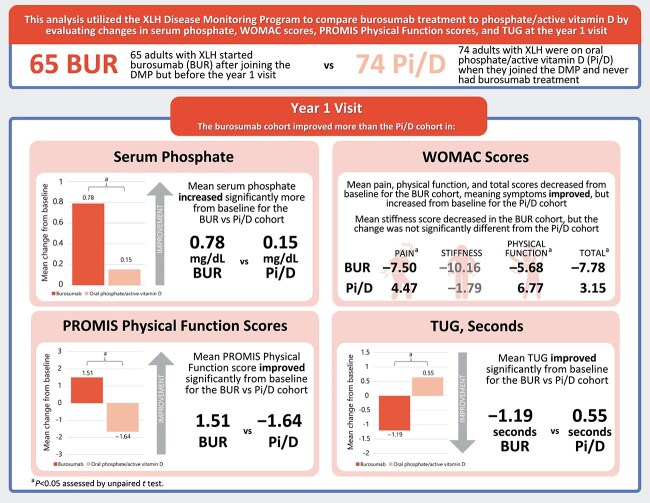

## Introduction

X-linked hypophosphatemia (XLH) results from loss-of-function variants in the phosphate-regulating endopeptidase homolog X-linked (*PHEX*) gene.^1–3^ Patients with XLH have elevated or inappropriately normal levels of the phosphate-regulating hormone FGF23 in the context of renal phosphate wasting.^1^^,^^2^^,^^4^^,^^5^ Chronic renal phosphate wasting leads to the development of rickets and short stature in children, and persistent osteomalacia in adults.^1–3^^,^^6^ Adults with XLH have a lifelong burden of disease, with an increased risk of osteomalacic fractures that heal poorly and other musculoskeletal manifestations, including osteoarthritis, enthesopathies, and spinal stenosis, contributing to pain, stiffness, and functional limitations such as impaired mobility.^3^^,^^4^^,^^7–9^

Burosumab is a fully human monoclonal Ab that neutralizes FGF23 and is approved for the treatment of XLH.^2^^,^^10^ By binding FGF23, burosumab improves phosphate reabsorption in the proximal renal tubules and also increases the production of 1,25(OH)_2_D.^2^^,^^11–13^ Treatment with burosumab has also demonstrated improved healing of osteomalacic fractures compared to placebo at week 24 of the pivotal randomized phase 3 study in adults with XLH.^11^ Additionally, in a single-arm, phase 3 study, burosumab significantly improved osteomalacia as measured by histologic and histomorphometric parameters after 48 wk of treatment.^2^

In addition to normalization of serum phosphate and improvement in osteomalacia, treatment efforts in adults are often aimed at addressing the symptomatic features of XLH, including pain reduction and promotion of fracture healing.^1^^,^^9^^,^^14^ Oral phosphate and active vitamin D (Pi/D) has been used to treat symptomatic adults with XLH, and has shown improvement in pain and osteomalacia.^1^^,^^9^^,^^15^ The continuation of Pi/D in adults is debated and not universal due to questions regarding benefits of long-term Pi/D relative to the magnitude of the risks (hypercalciuria, hyperparathyroidism, and nephrocalcinosis).^1^^,^^3^^,^^7^^,^^16^ Clinical practice recommendations for XLH management state that symptomatic adults (eg, those with biochemical/clinical manifestations of osteomalacia, musculoskeletal pain/stiffness) should receive Pi/D; however, studies report that only approximately 50% or fewer of adults are treated with Pi/D at any one time.^4^^,^^17^ Now that burosumab is available, it has been recommended for adults with XLH having clinical symptoms such as persistent bone and/or joint pain, restricted activity, and osteomalacia-related fractures (pseudofractures/active fractures), and in patients who are intolerant of or have had an inadequate musculoskeletal response to Pi/D otherwise.^4^^,^^9^^,^^16^

In the aforementioned pivotal phase 3 study, a significantly greater proportion of patients receiving burosumab achieved a mean serum phosphate concentration above the lower limit of normal (LLN; defined as 2.5 mg/dL) compared with placebo (94.1% vs 7.6%, *p* < .001) across midpoint intervals between baseline and week 24.^11^ In addition, there was a numerical improvement in Western Ontario McMaster Universities Osteoarthritis (WOMAC) index stiffness, and physical function, as well as Brief Pain Inventory-Short Form (BPI-SF) worst pain score at week 24; however, significant improvement was only observed in the stiffness subscale in patients receiving burosumab compared with the placebo-treated group.^11^ In an open-label extension study, mean serum phosphate remained at or above the LLN in 83.8% of burosumab-treated patients from 24 to 48 wk.^18^ By week 48, significant improvements from baseline in Brief Pain Inventory-Short Form (BPI-SF) worst pain score as well as WOMAC subscales for stiffness and physical function were observed in patients who received burosumab throughout the study period.^18^ Patient-reported outcomes assessed at 96 wk in an open-label extension study of the randomized phase 3 study showed that patients receiving burosumab had significant improvements from baseline in all WOMAC scores (pain, stiffness, physical function, and total).^19^

Whereas a randomized controlled trial of burosumab vs Pi/D in children with XLH demonstrated superiority of burosumab,^20^ no such trial in adults has compared burosumab to Pi/D. Therefore, this analysis utilized prospective, observational data to evaluate the real-world effectiveness of burosumab compared to Pi/D in adults with XLH.

## Materials and methods

### Data source

The XLH Disease Monitoring Program (DMP; NCT03651505) is a prospective, multicenter, longitudinal, 10-yr observational outcomes program that was initiated on July 16, 2018 and is being conducted in the United States, Canada, and Latin America (Argentina, Brazil, Chile, Colombia). The XLH DMP is designed to characterize XLH disease presentation and progression and also assess the long-term safety and effectiveness of burosumab treatment across the lifespan. The eligibility criteria for the DMP are inclusive of both adults and pediatric patients diagnosed with XLH (based on clinical features including short stature or leg deformities and biochemical profile consistent with XLH, or confirmed *PHEX* variant in the patient or in a family member) regardless of treatment status. Due to the real-world nature of the XLH DMP, patients could be on or off any XLH treatment at any time. Demographic, physiologic, disease severity, and progression data (including disease-related comorbidities) are collected via in-clinic assessments and patient phone interviews at study sites. Biochemical data were processed by a central laboratory for consistency.

For this analysis, adults enrolled in the DMP from study initiation to December 2022 were included. The complete safety data on 453 adults enrolled in the DMP, up to February 28, 2023, is available through the 2023 DMP Annual Report to the FDA. The main objective of the DMP safety review is to identify whether burosumab affects the risk of nephrocalcinosis, renal failure, spinal stenosis, pregnancy and lactation risks, or neonatal outcomes.

### Study population

**Figure 1:**
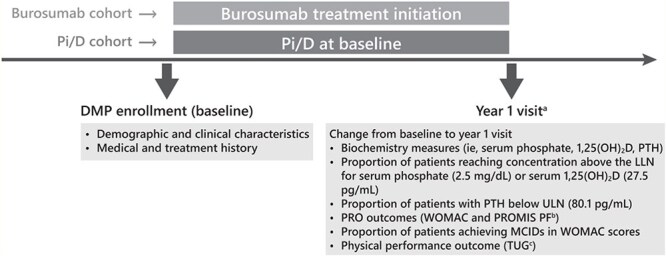
Study design. Abbreviations: 1,25(OH)_2_D, 1,25-dihydroxyvitamin D; DMP, Disease Monitoring Program; LLN, lower limit of normal; MCID, minimal clinically important difference; Pi/D, oral phosphate/active vitamin D; PROMIS PF, Patient-Reported Outcomes Measurement Information System Physical Function; PTH, parathyroid hormone; TUG, timed up and go; ULN, upper limit of normal; WOMAC, Western Ontario and McMaster Osteoarthritis Index. ^a^Due to the real-world nature of this study, the duration from baseline to the year 1 visit, and the duration from burosumab initiation to the year 1 visit may not be exactly 1 yr. ^b^PROMIS PF is a clinical tool that measures a patient’s physical function through a scale of activities of daily living and assesses the upper and lower extremities and central body regions. ^c^TUG is a clinical test that assesses fall risk by having the patient rise from a standard armchair, walking 3 m at a comfortable pace, turning around, walking back, and sitting down again. TUG times <15 s predict decreased fall risk.

This analysis included 2 cohorts of adults (age ≥ 18 yr) with XLH who either initiated burosumab after DMP study entry (baseline) but before the year 1 visit or reported being on Pi/D at DMP study entry and never received subsequent burosumab treatment (Figure 1). Patients were excluded if they had previously received burosumab treatment before enrolling in the DMP or if they did not complete the year 1 visit.

### Baseline characteristics and treatment outcomes

Baseline demographics and clinical characteristics, as well as medical and treatment history, were assessed and compared between the 2 cohorts.

Treatment information was ascertained from self-reported dosing records for the burosumab cohort and concomitant medication records for the Pi/D cohort. For the burosumab cohort, treatment duration was ascertained by subtracting the first dose date from the year 1 visit date. In addition, the duration between the last dose preceding the year 1 visit and the year 1 visit date was recorded in order to capture the variability of the year 1 visit measurement, which can fall within any date during a dosing cycle. For the Pi/D cohort, both Pi/D medication type and any discontinuation reported by patients following the baseline visit but before the year 1 visit were determined.

### Outcomes

Change from baseline in biochemistry (serum phosphate, 1,25[OH]_2_D, PTH), as well as the proportion of patients with serum phosphate concentration and 1,25(OH)_2_D above the LLN (defined as 2.5 mg/dL and 27.5 pg/mL, respectively) were assessed at baseline and the year 1 visit. Additionally, the proportion of patients reaching normal levels of PTH (below the upper limit of normal [ULN] of 80.1 pg/mL) at baseline and the year 1 visit was determined. Change from baseline to the year 1 visit in PROs (WOMAC pain, stiffness, physical function, and total scores and the Patient-Reported Outcomes Measurement Information System Physical Function [PROMIS PF] instrument) were evaluated. The proportion of patients achieving an improvement greater than the minimal clinically important difference (MCID) in WOMAC scores was also determined. The MCID is used to measure the smallest amount of meaningful change that is perceived by patients who have XLH.^21^^,^^22^ In this analysis, the MCID for WOMAC pain, stiffness, physical function, and total scores were >11, >10, >8, and >10, respectively, based on validation of the WOMAC in adults with XLH.^23^ Change in physical performance was assessed using the Timed Up and Go (TUG) test, a simple assessment of mobility, balance, and fall risk performed by timing (in seconds) how long it takes for a patient to rise from a standard armchair, walk 3 m at a comfortable pace, turn around, walk back, and sit down again.^24^^,^^25^

### Statistical analysis

Descriptive analyses were performed to summarize baseline characteristics, comorbidities, laboratory data, and PROs for the 2 cohorts. Continuous variables were expressed as mean and SE, while categorical variables were presented as the number of patients and proportions. All statistical tests were performed using a 2-tailed test, and a *p*-value of ≤.05 was considered statistically significant. Differences in continuous variables were assessed using unpaired *t* tests and proportions with chi-square tests.

PROs were assessed utilizing WOMAC and PROMIS PF scoring. Scoring for WOMAC is a sum metric normalized from 0 to 100, representing the percent of the maximum score, where 0 is the best health state and 100 the worst.^26–28^ The PROMIS PF is a clinical tool that measures a patient’s physical ability through scaled activities of daily living.^29^ A PROMIS PF score of 50 is equivalent to the results from the average US population, with lower scores representing decreased physical ability.^30^

Inverse probability of treatment weighting was employed to reduce potential measured confounding due to baseline differences between cohorts.^31^^,^^32^ As a first step, the probability of being exposed to a treatment (propensity score) was estimated using a logistic regression model, where treatment status was regressed on observed characteristics.

For this analysis, baseline demographic and clinical characteristics were used in the calculation of propensity scores. Additional details on propensity score calculation methodology are available in the Supplementary Methods. The estimated propensity score represented the probability of receiving one of the treatments being compared, given the measured covariates. The weight for each patient was calculated as the inverse of the probability of receiving treatment.

Missing covariate values were imputed using the Multiple Imputation with Fully Conditional Specification method, also known as imputation by chained equations or sequential generalized regression. Although this regression imputation model was used to impute the missing values for the continuous variables, it should be noted that no missingness was observed in categorical variables of interest. The imputations were performed 5 times and further propensity scores were generated for each of the 5 imputed complete datasets. Propensity scores were averaged across the 5 imputed complete datasets to generate average treatment effect on the treated (ATT) weights, which were used to determine the outcomes in a cohort of patients who received burosumab treatment in clinical practice.^33^ Average treatment effect weights were equal to 1 for the burosumab cohort and *p*/(1 − *p*) for the Pi/D cohort, where p is equal to the propensity score or probability of being in a burosumab cohort for the Pi/D cohort patients.^33^ Weights were truncated at the fifth and 95th percentile to address issues that can potentially surface due to very large weights.^31^^,^^33^

Standardized mean differences (SMDs) were reported before and after inverse probability of treatment weighting for baseline characteristics to show the balance improvement of the cohorts.^34^ While no fixed threshold was set for SMD, a value of <0.1 was considered a good indicator of balanced covariates, whereas SMD >0.25 was considered an indicator of remaining imbalance.

### Additional analyses

Patients in the burosumab cohort were not required to be on Pi/D at baseline. In order to account for any potential impact of prior Pi/D treatment on results, a scenario analysis was conducted, where outcomes for patients in the burosumab cohort were stratified according to Pi/D status at baseline.

Due to real-world differences in burosumab access, most patients treated with Pi/D in the DMP were from Latin American countries, whereas the majority of those in the burosumab cohort were from the United States and Canada. To address this limitation, an additional analysis was conducted to assess outcomes restricting the patient population to those in the United States and Canada only.

## Results

### Baseline characteristics

A total of 139 adult patients (burosumab, *n* = 65; Pi/D, *n* = 74) were included in this study after eligibility criteria were applied (Table 1). Most baseline characteristics were comparable between the 2 cohorts; however, before inverse probability of treatment weighting, the burosumab cohort was taller and heavier than the Pi/D cohort, with similar BMI. Country and ethnicity were significantly different due to the regions from which patients were enrolled in the XLH DMP, with patients from Latin American countries disproportionally not receiving burosumab treatment. Clinically, the burosumab cohort exhibited greater disease burden, evidenced by statistically significantly worse WOMAC (pain, stiffness, and total) scores and a higher proportion of medical conditions (genu valgum, depression) and pain medication at baseline.

**Table 1:** Baseline characteristics before and after inverse probability of treatment weighting.^a^^–^^c^

	**Before inverse probability of treatment weighting**				**After inverse probability of treatment weighting**			
**Characteristic**	**Burosumab** **(*N* = 65)**	**Pi/D** **(*N* = 74)**	***p-*value**	**SMD**	**Burosumab** **(*N* = 65.0)**	**Pi/D** **(*N* = 32.6)**	***p-*value**	**SMD**
**Age, mean years (SE)** ^**d**^	39.44 (1.61)	39.32 (1.79)	.960	0.01	39.44 (1.61)	38.46 (2.50)	.736	0.09
**Sex, *n* (%)** ^**d**^								
** Female**	48 (73.9)	59 (79.7)	.411	−0.14	48 (73.9)	24.7 (75.8)	.858	−0.05
** Male**	17 (26.2)	15 (20.3)			17 (26.2)	7.9 (24.2)		
**Race, *n* (%)** ^**d**^								
** White**	49 (75.4)	60 (81.1)	.139	−0.14	49 (75.4)	24.0 (73.4)	.909	0.04
** Non-White**	3 (4.6)	7 (9.5)		−0.19	3 (4.6)	2.3 (7.0)		−0.10
** Unknown/not reported**	13 (20.0)	7 (9.5)		0.30	13 (20.0)	6.4 (19.6)		0.01
**Ethnicity, *n* (%)**								
** Hispanic or Latino**	6 (9.2)	52 (70.3)	<.001	−1.60	6 (9.2)	16.3 (49.9)	<.001	−1.00
** Not Hispanic or Latino**	47 (72.3)	17 (23.0)		1.14	47 (72.3)	11.4 (34.9)		0.81
** Other**	12 (18.5)	5 (6.8)		0.36	12 (18.5)	5.0 (15.2)		0.09
**Country, *n* (%)**								
** Brazil**	3 (4.6)	28 (37.8)	<.001	−0.89	3 (4.6)	7.9 (24.1)	<.001	−0.58
** Canada**	11 (16.9)	5 (6.8)		0.32	11 (16.9)	5.0 (15.2)		0.05
** Chile**	0	22 (29.7)		−0.31	0	6.9 (21.1)		−0.11
** Colombia**	1 (1.5)	1 (1.4)		0.02	1 (1.5)	1.2 (3.7)		−0.13
** USA**	50 (76.9)	18 (24.3)		1.24	50 (76.9)	11.7 (35.8)		0.91
**Weight, mean kg (SE)** ^**d**^	73.84 (2.43)	66.27 (1.75)	.013	0.43	73.84 (2.43)	71.43 (3.12)	.560	0.16
**Height, mean cm (SE)** ^**d**^	153.30 (1.17)	147.35 (1.24)	.001	0.59	153.30 (1.17)	153.39 (3.00)	.973	0.03
**Body mass index, mean (SE)**	31.31 (1.06)	30.61 (0.84)	.604	0.09	31.31 (1.06)	31.10 (2.13)	.922	0.03
**Serum phosphate concentration (mg/dL), mean (SE)** ^**d**^ ^**,**^ ^**e**^	2.11 (0.04)	2.20 (0.05)	.223	−0.21	2.11 (0.04)	2.18 (0.13)	.516	−0.19
**1,25(OH)**_**2**_ **D (pg/mL), mean (SE)** ^**d**^ ^**,**^ ^**e**^	40.73 (2.24)	45.79 (2.40)	.134	−0.26	40.73 (2.24)	42.45 (2.81)	.650	−0.10
**PTH (pg/mL), mean (SE)** ^**d**^ ^**,**^ ^**e**^	83.40 (6.04)	88.41 (11.29)	.697	−0.06	83.40 (6.04)	81.84 (13.91)	.905	0.03
**WOMAC score, mean (SE)** ^**f**^								
** Pain^d^**	38.54 (2.77)	28.26 (2.96)	.013	0.43	38.54 (2.77)	34.23 (5.32)	.431	0.22
** Physical function^d^**	34.30 (3.03)	30.51 (3.21)	.395	0.15	34.30 (3.03)	27.55 (4.80)	.223	0.32
** Stiffness^d^**	52.12 (2.92)	38.70 (3.50)	.004	0.50	52.12 (2.92)	48.16 (5.51)	.489	0.20
** Total**	41.65 (2.68)	32.52 (3.25)	.001	0.35	41.65 (2.68)	36.65 (4.62)	.322	0.23
**PROMIS PF, mean (SE)** ^**d**^ ^**,**^ ^**g**^	40.67 (1.10)	42.03 (1.07)	.379	−0.15	40.67 (1.10)	42.52 (2.18)	.402	−0.24
**TUG, mean (SE)** ^**h**^	10.48 (0.83)	12.04 (1.08)	.255	−0.19	10.48 (0.83)	9.87 (0.45)	.623	0.09
**Bowing of legs, *n* (%)** ^**d**^	53 (81.5)	53 (71.6)	.170	0.23	53 (81.5)	24.7 (75.8)	.589	0.17
**Genu valgum, *n* (%)** ^**d**^	10 (15.4)	28 (37.8)	.003	−0.52	10 (15.4)	6.5 (19.9)	.619	−0.14
**Intoeing, *n* (%)** ^**d**^	33 (50.8)	31 (41.9)	.295	0.18	33 (50.8)	14.9 (45.6)	.678	0.12
**Osteoarthritis, *n* (%)** ^**d**^	30 (46.2)	30 (40.5)	.505	0.11	30 (46.2)	13.5 (41.5)	.707	0.11
**Enthesopathy/bone spurs/osteophytes, *n* (%)** ^**d**^	36 (55.4)	40 (54.1)	.875	0.03	36 (55.4)	15.0 (46.0)	.565	0.22
**Spinal cord compression, *n* (%)** ^**d**^	11 (16.9)	5 (6.8)	.061	0.32	11 (16.9)	3.2 (9.8)	.392	0.24
**Nontraumatic fracture/pseudofracture, *n* (%)** ^**d**^	22 (33.9)	25 (33.8)	.994	<0.01	22 (33.9)	7.8 (23.8)	.317	0.26
**Traumatic fracture, *n* (%)**	16 (24.6)	14 (18.9)	.415	0.14	16 (24.6)	9.9 (30.5)	.622	−0.16
**Number of fractures ever** ^**i**^ **, mean (SE)** ^**d**^ ^**,**^ ^**i**^	5.50 (2.31)	3.06 (0.50)	.311	0.15	5.50 (2.31)	3.64 (1.14)	.964	0.07
**Spinal surgery, *n* (%)** ^**d**^	5 (7.7)	1 (1.4)	.066	0.31	5 (7.7)	0.1 (0.4)	.032	0.38
**Tinnitus, *n* (%)** ^**d**^	20 (30.8)	18 (24.3)	.395	0.14	20 (30.8)	8.3 (25.4)	.628	0.14
**Hearing loss, *n* (%)** ^**d**^	18 (27.7)	17 (23.0)	.522	0.11	18 (27.7)	7.6 (23.3)	.683	0.12
**Hyperparathyroidism, *n* (%)** ^**d**^	15 (23.1)	17 (23.0)	.988	<0.01	15 (23.1)	9.7 (29.9)	.562	−0.18
**Nephrocalcinosis, *n* (%)** ^**d**^	9 (13.9)	15 (20.3)	.317	−0.17	9 (13.9)	4.2 (12.9)	.917	0.03
**Hypertension, *n* (%)** ^**d**^	14 (21.5)	8 (10.8)	.084	0.29	14 (21.5)	6.4 (19.8)	.866	0.05
**Headache, *n* (%)** ^**d**^	19 (29.2)	16 (21.6)	.302	0.17	19 (29.2)	8.4 (25.7)	.763	0.09
**Severe headache, *n* (%)** ^**d**^	15 (23.1)	13 (17.6)	.419	0.14	15 (23.1)	8.1 (24.8)	.881	−0.05
**Depression, *n* (%)** ^**d**^	19 (29.2)	2 (2.7)	<.001	0.77	19 (29.2)	2.2 (6.7)	.003	0.65
**Age at XLH diagnosis, mean years (SE)** ^**d**^	10.29 (2.15)	7.61 (1.20)	.278	0.19	10.29 (2.15)	9.25 (2.80)	.776	0.08
**History of Pi/D ever, *n* (%)**	57 (87.7)	74 (100.0)	.002	−0.53	57 (87.7)	32.6 (100.0)	.037	−0.53
**History of pediatric Pi/D, *n* (%)** ^**d**^	51 (78.5)	59 (79.7)	.854	−0.03	51 (78.5)	25.1 (77.0)	.889	0.03
**Pi/D at baseline, *n* (%)**	28 (43.1)	74 (100.0)	<.001	−1.63	28 (43.1)	32.6 (100.0)	<.001	−1.63
**Any pain medication at baseline, *n* (%)** ^**d**^	39 (60.0)	27 (36.5)	.006	0.48	39 (60.0)	15.1 (46.4)	.273	0.32
**Any opioid medication at baseline, *n* (%)** ^**d**^	8 (12.3)	6 (8.1)	.412	0.14	8 (12.3)	4.3 (13.0)	.935	−0.03

Abbreviations: Pi/D, oral phosphate/active vitamin D; PROMIS PF, Patient-Reported Outcomes Measurement Information System Physical Function; PTH, parathyroid hormone; SE, standard error; SMD, standard mean difference; TUG, Timed Up and Go; WOMAC, Western Ontario and McMaster Osteoarthritis Index; XLH, X-linked hypophosphatemia.

a Weighted n (%) is reported.

b Sandwich variance estimator is used to estimate SE.

c For inverse probability of treatment weighting, each patient is assigned a weight to make the cohort on average similar to the burosumab cohort, where each patient has a weight of 1.

d Patient characteristics, treatment and medical history included in the propensity score estimation.

e Normal ranges: serum phosphate, 2.5-4.5 mg/dL; 1,25(OH)_2_D, 18-72 pg/mL; PTH, 14-72 pg/mL.

f The WOMAC index is scored on a scale of 0-100. A higher WOMAC score indicates a worse outcome.^35^

g PROMIS PF score of 50 is equivalent to results from an average population.^30^

h A higher TUG score indicates more impairment in mobility and physical ability.^24^

i Includes traumatic and nontraumatic/pseudofractures.

After inverse probability of treatment weighting, the balance of characteristics between the burosumab and Pi/D cohorts was improved, demonstrated by the SMDs and *p*-values (Table 1); however, based on SMDs >0.25, some minor imbalances among covariates included in the propensity score estimation (specifically in nontraumatic fractures/pseudofractures, depression, spinal surgery, any pain medication at baseline, WOMAC physical function score) remained after adjustment.

### Treatment patterns

The year 1 follow-up visit occurred a median (interquartile range [IQR]; min, max) of 12.89 (11.97, 15.09; 9.57, 25.90) mo after their baseline visit. The mean (SE) time between baseline and the year 1 visit was similar between cohorts (burosumab, 13.81 [0.41] mo vs Pi/D, 14.22 [0.34] mo). Median (IQR; min, max) burosumab treatment duration was 9.34 (5.75, 11.67; 0.26, 23.04) mo. The treatment duration range varied due to some patients having their year 1 visit either very early or late after DMP enrollment and burosumab initiation. Additionally, the COVID pandemic severely impacted patient visit schedules. Ten (15.4%) patients received burosumab for less than 3 mo, 8 (12.3%) from 3 to 6 mo, and 47 (72.3%) for more than 6 mo. Median (IQR) time between the most recent burosumab dose before the year 1 visit and the measurements of the year 1 visit was 14 (7, 25) d, which may add variability to year 1 outcomes for the burosumab cohort given the outcomes were measured at various points within the 4-wk dosing cycle, or even later than the 4-wk time point if doses had been missed. Eight (12.3%) patients in the burosumab cohort reported no dose of burosumab after the year 1 visit, with a median (IQR) time from the last dose to the year 1 visit of 3.53 (0.61, 6.34) mo, potentially due to discontinuation or doses not being reported.

Self-reported Pi/D treatments in the PiD cohort included oral phosphate, calcitriol, cholecalciferol, other vitamin D and analogs, and unspecified forms of vitamin D. Calcitriol had the lowest discontinuation rate (approximately 14% of patients discontinued a median [IQR] of 4.80 [3.58, 7.82] mo before the year 1 visit), whereas 33%-43% of patients discontinued all other Pi/D treatments (Table S1).

### Biochemistry at the year 1 visit

**Figure 2:**
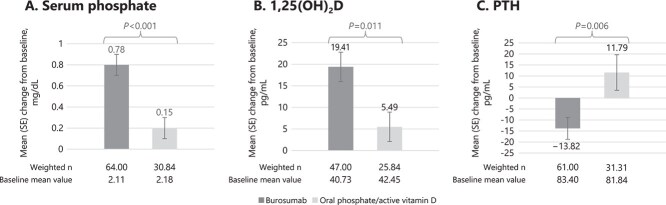
Biochemistry measures after inverse probability of treatment weighting: A. Serum phosphate, B. 1,25(OH)_2_D, C. PTH. Abbreviations: 1,25(OH)_2_D, 1,25-dihydroxyvitamin D; PTH, parathyroid hormone; SE, standard error.

**Figure 3:**
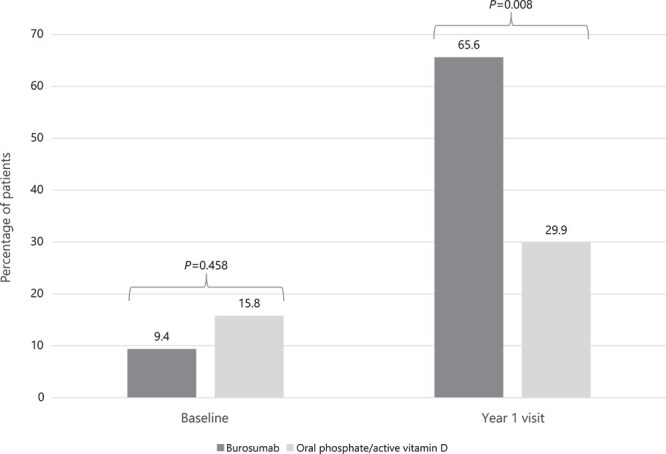
Proportion of patients in the burosumab vs Pi/D cohorts with serum phosphate concentrations >LLN^a^ at baseline and year 1 visit after inverse probability of treatment weighting. Abbreviations: LLN, lower limit of normal; Pi/D, oral phosphate/active vitamin D. ^a^LLN defined as serum phosphate concentration of 2.5 mg/dL.

After inverse probability of treatment weighting, there was a significantly greater improvement from baseline in mean (SE) serum phosphate concentration observed in the burosumab cohort when compared with the Pi/D cohort (0.78 [0.08] mg/dL vs 0.15 [0.14] mg/dL, *p* < .001; Figure 2A; Table S2). At baseline, the proportion of patients with serum phosphate concentrations greater than the LLN was similar between the burosumab and Pi/D cohorts (9.4% vs 15.8%, *p =* .458; Figure 3). At the year 1 visit, a significantly higher proportion of patients had serum phosphate concentrations greater than the LLN in the burosumab cohort compared with the Pi/D cohort (65.6% vs 29.9%, *p* = .008; Figure 3).

Similarly, improvements in serum 1,25(OH)_2_D and PTH at the year 1 visit were significantly greater for the burosumab cohort than the Pi/D cohort (serum 1,25(OH)_2_D: 19.41 [3.39] pg/mL vs 5.49 [3.43] pg/mL, *p* = .011; PTH: −13.82 [5.00] pg/mL vs 11.79 [8.10] pg/mL, *p* = .006; Figure 2B and C). Similar proportions of patients in the burosumab and Pi/D cohorts had a serum 1,25(OH)_2_D above the LLN at baseline (85.1% vs 89.4%; *p* = .600) and at the year 1 visit (93.6% vs 90.2%; *p* = .600). The proportion of patients with values below the ULN for PTH at baseline in the burosumab cohort was 55.7% vs 40.2% in the Pi/D cohort. A higher proportion of patients had normal PTH at year 1 in the burosumab cohort compared to the Pi/D cohort (70.5% vs 56.0%; *p* = .228).

In the unweighted groups, improvement in serum phosphate was similar to the weighted cohorts, with a significantly greater improvement with burosumab compared with Pi/D (0.78 [0.08] mg/dL vs 0.14 [0.06] mg/dL; *p* < .0001, Table S3). Additionally, the baseline proportion of patients with serum phosphate concentrations greater than the LLN was similar between the burosumab and Pi/D cohorts (9.4% vs 12.7%, *p* = .542), and a significantly higher proportion of patients had serum phosphate concentrations greater than the LLN in the burosumab cohort compared with the Pi/D cohort (65.6% vs 23.9%, *p* < .0001) at the year 1 visit.

In the unweighted treatment groups, serum 1,25(OH)_2_D improved significantly with burosumab vs Pi/D (19.41 [3.39] pg/mL vs −1.14 [1.89] pg/mL; *p* < .0001). Serum PTH improved for both treatment cohorts in the unweighted groups, but greater improvement was observed in the burosumab cohort compared with the Pi/D cohort (−13.82 [5.00] pg/mL vs −1.39 [4.50] pg/mL; *p* = .066).

Prior to weighting, similar proportions of patients in the burosumab and Pi/D cohorts had a serum 1,25(OH)_2_D above the LLN at baseline (85.1% vs 86.9%; *p* = .791) and at the year 1 visit (93.6% vs 88.5%; *p* = .365). These values were comparable to the weighted groups. The proportion of patients with values below the ULN for PTH at baseline was higher in the unweighted vs weighted Pi/D cohort (60.6% vs 40.2%), but the proportion of patients in the unweighted burosumab cohort mirrored the weighted cohort (both 55.7%). Before weighting, a similar proportion of patients had normal PTH at year 1 with burosumab vs Pi/D (70.5% vs 66.2%; *p* = .676).

### Patient-reported outcomes and TUG physical performance at the year 1 visit

**Figure 4:**
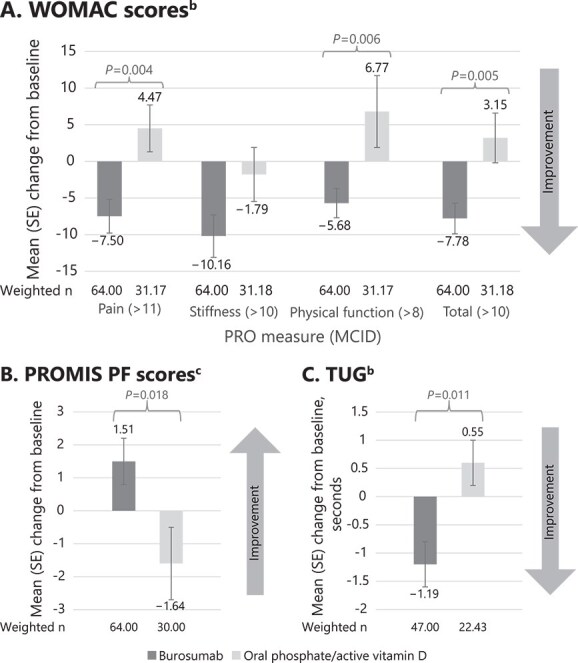
Mean (SE) change from baseline in PRO scores and physical performance outcomes after inverse probability of treatment weighting^a^: A. WOMAC, B. PROMIS PF, C. TUG. Abbreviations: MCID, minimal clinically important difference; PRO, patient-reported outcome; PROMIS PF, Patient-Reported Outcomes Measurement Information System Physical Function; SE, standard error; TUG, Timed Up and Go; WOMAC, Western Ontario and McMaster Osteoarthritis Index. ^a^Assessed using unpaired *t-*tests. ^b^A negative change is consistent with improvement. ^c^A positive change is consistent with improvement.

**Figure 5:**
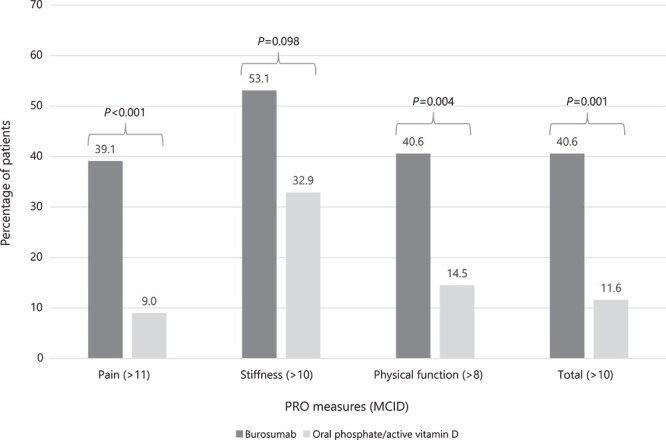
Proportion of patients achieving improvement > MCID WOMAC scores at year 1 visit after inverse probability of treatment weighting. Abbreviations: MCID, minimal clinically important difference; PRO, patient-reported outcome; WOMAC, Western Ontario and McMaster Osteoarthritis.

At the year 1 visit, differences in mean (SE) change from baseline in 3 of the 4 tested WOMAC scores improved significantly for the burosumab vs Pi/D cohort (pain: −7.50 [2.34] vs 4.47 [3.23], *p* = .004; physical function: −5.68 [1.96] vs 6.77 [4.85], *p* = .006; total: −7.78 [2.06] vs 3.15 [3.37], *p* = .005) (Figure 4A; Table S4). The change in stiffness score was numerically greater for the burosumab cohort when compared with Pi/D, but was not statistically significant (mean [SE] change from baseline of −10.16 [2.85] vs −1.79 [3.68], *p* = .086). A significantly greater proportion of patients achieved the WOMAC MCID for pain (39.1% vs 9.0%; *p* < .001), physical function (40.6% vs 14.5%; *p* = .004), and total score (40.6% vs 11.6%; *p* = .001) in the burosumab cohort compared with the Pi/D cohort (Figure 5). The proportion of patients achieving the MCID for WOMAC stiffness was greater for the burosumab cohort vs the Pi/D cohort, but did not reach statistical significance (53.1% vs 32.9%; *p* = .098).

At the year 1 visit, mean (SE) change from baseline in both PROMIS PF score (1.51 [0.73] vs −1.64 [1.11]; *p* = .018) and TUG (−1.19 [0.42] seconds vs 0.55 [0.43] s; *p* = .011) improved significantly in the burosumab cohort compared with the Pi/D cohort (Figure 4B and C; Table S4).

In the unweighted groups, differences in mean (SE) change from baseline in 3 of the 4 tested WOMAC scores were statistically significant at the year 1 visit with burosumab vs Pi/D (pain: −7.50 [2.34] vs 2.25 [1.98], *p* = .002; stiffness: −10.16 [2.85] vs −0.69 [3.05], *p* = .026; total: −7.78 [2.06] vs 0.44 [2.47], *p* < .0001; Table S3). This differed from the weighted groups, where significant improvements with burosumab vs Pi/D were observed in WOMAC physical function, but not stiffness. Also, a significantly greater proportion of patients in the unweighted groups achieved the WOMAC MCID only for pain (39.1% vs 12.7%; *p* < .001) and total (40.6% vs 19.4%; *p* = .007) scores in the burosumab cohort compared with the Pi/D cohort (Figure S1). In contrast to the weighted groups, TUG score was similar for the unweighted treatment cohorts (burosumab: −1.19 [0.42] s vs Pi/D: −0.15 [0.99] s; *p* = .334).

In summary, the statistical significance of biochemistry (serum phosphate and 1,25(OH)_2_D) and WOMAC (pain and total) outcomes was consistent between pre- and postinverse probability of treatment weighting adjustment (Tables S2-S4**)**, indicating robustness of the results despite potential confounding.

### Additional analyses

Twenty-eight of the 65 patients in the burosumab cohort reported receiving Pi/D at baseline. There were no significant differences between patients in the burosumab cohort who were treated with Pi/D at baseline compared to those who were not for baseline age, sex, weight, height, BMI, biochemistry measures, PROs, and TUG scores (Table S5). However, significant differences were observed in race, ethnicity, and country; a higher proportion of patients not receiving Pi/D at baseline were non-Hispanic and from the United States. There were no significant differences in the year 1 outcome measures between patients receiving Pi/D at baseline and patients who were not on Pi/D at baseline (Table S6), suggesting that baseline Pi/D treatment status does not substantially affect the effectiveness of burosumab treatment.

After restricting to patients from the United States and Canada only without weighting adjustment, 61 patients remained in the burosumab cohort and 23 in the Pi/D cohort. Baseline demographics were similar between the 2 cohorts, except for a significantly higher mean age in the Pi/D cohort and worse PRO scores in the burosumab cohort (Table S7). Outcomes were largely comparable to the main analysis with the burosumab cohort showing significant improvements in biochemistry, WOMAC (pain, physical function, total), and TUG scores (Table S8). A numerical improvement in WOMAC stiffness and PROMIS PF scores was observed in the burosumab cohort, but neither PRO outcome reached statistical significance.

### Safety

Overall, the available data on renal health, including nephrocalcinosis, is consistent with the trends observed during burosumab clinical studies. Only one nonserious adverse event (AE; preferred term: blood creatinine increased, related) and one serious AE (preferred term: acute kidney injury, not related) suggestive of renal impairment were reported in adult patients. Additionally, nonserious burosumab-related AEs of nephrocalcinosis were reported in 4 adults. The renal laboratory assessments, including serum creatinine, estimated glomerular filtration rate (eGFR), and urine protein to creatinine ratio, were overall within the normal range.

A total of 10 serious AEs involving spinal stenosis were reported in 9 adults. All events were assessed as not related since spinal stenosis was present prior to enrollment in the XLH DMP.

A total of 27 patients experienced pregnancy during the XLH DMP, from which 24 live births were reported. Most patients discontinued burosumab upon knowledge of the pregnancy; however, in approximately 6 patients, discontinuation information was not provided or the patient was lost to follow-up. A total of 6 serious AEs related to pregnancy were reported and all were assessed as not related to burosumab.

A patient’s infant was exposed to burosumab via lactation. No AEs were reported in either the patient or the infant.

Upon review of the annual XLH DMP data, no new safety concerns were identified, and there was no evidence to suggest a risk of nephrocalcinosis, renal failure, spinal stenosis/spinal cord compression, pregnancy and lactation risks, or neonatal outcomes with burosumab treatment. Overall, the benefit-risk profile of burosumab remains positive in adult patients with XLH.

## Discussion

Evidence comparing outcomes in adults with XLH receiving burosumab treatment vs Pi/D has been lacking, largely due to the absence of Pi/D comparator arms in randomized clinical trials.^36^ In a recently published systematic literature review that presented outcomes of treatment with burosumab and Pi/D in patients with XLH, no head-to-head studies of burosumab vs Pi/D in adults were identified.^36^ To the best of our knowledge, the current study is the first comparative study of burosumab vs Pi/D in adults with XLH, and results showed that burosumab treatment was associated with significant improvements in biochemical parameters, PROs, and mobility, compared with Pi/D.

The use of inverse probability of treatment weighting in real-world studies allows for the analysis of more similar populations by balancing potential measured confounders between study cohorts.^31^^,^^32^ Additionally, it should be noted that the use of inverse probability of treatment weighting has been increasingly used in the literature,^32^ including to estimate average treatment effects in various settings that are focused on clinical learnings from real-world data.^37–41^ The National Institute for Health and Care Excellence (NICE) developed a framework to provide guidance on best practices when conducting real-world evidence studies and has cited inverse probability of treatment weighting as a means to address handling of missing data, selection bias, and observed confounders.^42^ Also, the FDA provided nonbinding recommendations in 2023 titled “Adjusting for Covariates in Randomized Clinical Trials for Drugs and Biological Products Final Guidance for Industry,” which stated that inverse probability of treatment weighting is a reliable method of covariate adjustment for unconditional treatment effects.^43^^,^^44^

In the current study, baseline characteristics and medical and treatment history (including those baseline factors used for propensity score estimation) were more balanced after inverse probability of treatment weighting; however, substantial differences between cohorts prohibited balancing of certain characteristics (country, ethnicity) that were not included in the propensity score estimation model. It is of note that even after random treatment assignment in randomized controlled trials, there may be remaining covariate imbalances purely by chance.^43^^,^^45^^,^^46^ The current analysis includes almost 40 baseline patient factors and, probabilistically, it can be expected to observe imbalances in approximately 2 factors over and above factors not included in the propensity score model. In this study, the Pi/D cohort had less disease burden (fewer patients with bowing of the legs and hearing loss) when compared with the burosumab cohort at baseline. Patients with greater disease burden were more likely to receive treatment with burosumab, which may be a source of bias and could potentially affect response to treatment; however, this point highlights the importance of utilizing inverse probability of treatment weighting to adjust for confounding. Additionally, the results were broadly consistent before and after adjustment, strengthening the conclusions of the analysis.

In the weighted cohorts, burosumab treatment was associated with significant improvement from baseline in serum phosphate, 1,25(OH)_2_D, and PTH when compared with Pi/D treatment. Notably, in the burosumab group, mean PTH decreased while it increased in the Pi/D group at the year 1 visit. In conjunction with FGF23 and 1,25(OH)_2_D, PTH plays an essential role in phosphate homeostasis by increasing efflux of calcium and phosphate from bone.^5^^,^^47^ Since PTH secretion is upregulated by low serum calcium and elevated serum phosphate levels,^5^^,^^47^^,^^48^ one may have expected to see a PTH-stimulatory effect in response to the increase in serum phosphate observed in this analysis. These data suggest that the release of FGF23 inhibition on 1,25(OH)_2_D synthesis by burosumab may outweigh the PTH-stimulatory effect of improved serum phosphate levels and thereby play a role in controlling PTH secretion during the course of FGF23 neutralization therapy.

Musculoskeletal pain and physical limitations are well-known burdens of XLH, which contribute to a patient’s diminished quality of life.^7^ In this analysis, a significantly greater reduction in WOMAC pain, physical function, and total scores was observed in the burosumab cohort compared with the Pi/D cohort at the year 1 visit. Additionally, a higher proportion of burosumab-treated patients achieved an improvement greater than the MCID, the smallest meaningful change perceived by a patient.^21^^,^^22^ PROMIS PF scores were also significantly improved in the burosumab cohort compared with the Pi/D cohort, providing further evidence for an improvement in physical function. There was a significant decrease in TUG in the burosumab cohort compared with the Pi/D cohort at the year 1 visit, indicating an improvement in physical mobility and a decrease in predicted fall risk.^24^^,^^25^ In addition to the relatively static biochemical parameters in the Pi/D cohort, outcomes related to symptoms and physical functioning worsened (increases in WOMAC pain, physical function, and total scores and in TUG time, as well as a decrease in PROMIS PF), suggesting limitations of Pi/D treatment in XLH.

Prior to the approval of burosumab, medical management of children with XLH focused on the use of Pi/D, whereas consensus regarding Pi/D use in adults was lacking.^1^^,^^3^ The utilization of Pi/D can be hindered by adverse events (increased risk of nephrocalcinosis and hyperparathyroidism) associated with oral phosphate supplementation as well as the need for frequent daily dosing.^1^^,^^3^^,^^4^^,^^9^^,^^16^ Although reasons for stopping Pi/D were not provided in the current study, discontinuation rates were relatively high, with approximately 33% of patients discontinuing oral phosphate at a median (IQR) of 6.41 (4.49, 8.15) mo prior to the year 1 visit. Additionally, active vitamin D supplementation was discontinued in approximately 14%-43% of patients at a median of 4.80-7.13 mo prior to the year 1 visit. In comparison, treatment discontinuation potentially occurred among 12.3% of patients in the burosumab cohort who had no recorded dose after the year 1 visit and received the last dose at a median (IQR) of 3.53 (0.61, 6.34) mo preceding the year 1 visit.

Patients included in the burosumab cohort had comparable baseline characteristics (age, race, and BMI) to patients in the pivotal phase 3 clinical trial of burosumab treatment in adults with XLH (NCT02526160); however, patients in the DMP exhibited higher mean baseline serum phosphate and 1,25(OH)_2_D and lower mean PTH concentrations, along with lower WOMAC (pain, stiffness, physical function) scores compared to the phase 3 clinical trial participants, indicating less severe disease.^11^^,^^18^^,^^19^ These baseline differences between the burosumab cohort in the current study and the pivotal phase 3 study are likely due to more stringent inclusion criteria for the clinical trial. The phase 3 study, for example, required patients to have a BPI “worst score” ≥4 on a scale of 0 (indicating no pain) to 10 (signifying worst pain),^11^^,^^18^^,^^49^ whereas the DMP accepted all patients with confirmed XLH irrespective of pain severity.

Despite baseline differences in study populations, the serum phosphate response to burosumab in this study was comparable to that observed in the phase 3 clinical trial.^18^ In the phase 3 study, serum phosphate increased from a baseline mean (SD) value of 2.0 (0.30) mg/dL by a least squares mean (95% CI) of 0.9 mg/dL (95% CI, 0.7-1.0) at 46 wk (mid-point of dose cycle, peak timepoint) and by 0.4 mg/dL (95% CI, 0.2-0.5) at 48 wk (end of dose cycle, trough timepoint).^18^ In the current study, the change from baseline in serum phosphate improved by a mean (SE) of 0.78 (0.08) mg/dL at the year 1 visit, despite a variable time of serum phosphate measurement following burosumab dosing. Furthermore, the proportion of patients with a serum phosphate concentration above the LLN was significantly greater in the burosumab cohort than in the Pi/D cohort. However, the proportion of patients achieving serum phosphate normalization in the DMP burosumab cohort (65.6%) was lower than that of the clinical trial (83.8% at week 48),^18^ potentially due to variation in the timing of serum phosphate measurement or treatment adherence in the DMP.

At week 48 of the clinical trial, WOMAC pain, stiffness, and physical function scores decreased significantly from baseline (least squares mean [SE] difference of −10.00 [2.55]; *p* < .05, −16.03 [3.32]; *p* < .001, and −7.76 [2.15]; *p* < .001, respectively) with burosumab.^18^^,^^19^ After inverse probability of treatment weighting in the DMP study, WOMAC pain, physical function, and total scores improved significantly from baseline, while WOMAC stiffness showed a numerical decrease for the burosumab cohort. However, the magnitude of benefit observed in the DMP was smaller compared to the clinical trial, possibly due to having a population with milder disease and therefore a lower capacity to benefit; patients in the DMP had better baseline scores than participants in the clinical trial for both pain (mean [SE]: 38.54 [2.77] vs mean [SD]: 50.7 [18.0]) and physical function (mean [SE]: 34.30 [3.03] vs mean [SD]: 50.8 [19.7]).^18^^,^^19^

The 6-min walk test (6MWT) has been utilized in phase 3 clinical trials to measure functional capacity in patients with XLH^18^^,^^19^^,^^50^ and involves having a patient walk a premeasured course continuously for 6 min.^19^^,^^50^ In the phase 3 trial, mean (SD) 6MWT, which was 356.8 (109.5) m at baseline, increased by 30.5 (6.93) m (*p* < .001) after 48 wk of burosumab treatment.^18^^,^^19^ In the DMP analysis, mobility assessed by TUG test improved significantly from baseline to the year 1 visit, demonstrating that the TUG physical performance test could potentially lend itself as a useful clinical tool for quick assessment of patients vs having to set up a 6MWT course in a clinic setting.

The current analysis had a number of limitations. Due to the real-world nature of the DMP, the duration from baseline to the year 1 visit was not uniform across all patients. Additionally, the interval between DMP enrollment and the initiation of burosumab treatment differed among patients, leading to varying treatment durations (average treatment duration was less than 1 yr) between baseline and the year 1 visit. Time of dosing to the time of serum phosphate measurement also varied by patient; serum phosphate measurement could occur at anytime in the dosing interval. This variation in both duration of treatment and timing of last dose could also have affected the results of other tests performed at the year 1 study visit.

The DMP provides Pi/D treatment information based on patient-reported use of oral phosphate, simple vitamin D (eg, cholecalciferol), and active vitamin D (eg, calcitriol). There was likely variability in phosphate supplementation dose, the active (or simple) vitamin D dose, and inconsistency in Pi/D adherence. Additionally, due to DMP categorization of Pi/D treatment reporting, there is potential overlap of vitamin D treatment categories (eg, the category of “vitamin D and analogs” or “vitamin D not otherwise specified” may include cholecalciferol and/or calcitriol). Unfortunately, these categories do not provide specific information on which form of vitamin D was used, which differs from the clinical trial setting where the form of vitamin D was recorded more precisely.^20^

Data were collected at scheduled visits per the DMP protocol; however, missing data may occur due to the real-world nature of the program. The DMP database has limitations in the types of data collected (eg, serum alkaline phosphatase is collected for pediatric patients only and serum calcium levels are not available for all patients). Unavailability of certain data can also make diagnosis of comorbidities difficult. For example, missing calcium values make it impossible to evaluate hyperparathyroidism and determine if it is classified as primary, secondary, or tertiary.

The overall analysis and the 2 additional analyses had small sample sizes; however, the sample sizes were sufficient to demonstrate statistically significant differences in change from baseline for many outcomes. Despite inverse probability of treatment weighting adjustment, significant differences remained in ethnicity, with more Hispanic or Latino patients in the Pi/D cohort when compared to the burosumab cohort. In line with the difference in ethnicity, a significantly higher representation from Latin America was present in the Pi/D cohort and from the US in the burosumab cohort. These differences resulted in a significant variation in country and ethnicity distribution between cohorts and highlight the discrepancy in accessibility of burosumab treatment between countries. Although countries were unbalanced, the additional analysis restricting to the United States and Canada patients showed comparable results.

Over half of the patients in the burosumab cohort were not on Pi/D at baseline, but the additional analysis performed showed that results are comparable when restricted to patients treated with Pi/D at baseline in the burosumab cohort. These results suggest that, over a 1-yr period, the changes in biochemistry, PRO scores, and physical outcomes are not affected by the baseline Pi/D status.

Lastly, although remaining imbalances after application of inverse probability of treatment weighting were considered generally minor and unlikely to have a significant confounding effect on outcomes, it cannot be guaranteed that unobserved confounders do not exist.

Despite these limitations, the results from this analysis are broadly consistent with those of the pivotal clinical trial and extension trials.^11^^,^^18^^,^^19^

## Conclusion

In this real-world analysis of adult patients with XLH, burosumab was associated with improvement in biochemistry measures, PROs, and TUG physical performance when compared to treatment with Pi/D. Additional studies are required to determine if the results of this analysis are sustained over an extended follow-up period.

## Supplementary Material

Revised_Supplemental_Information_FilledOut_zjaf063

## Data Availability

The data that support the findings of this study are available from Kyowa Kirin, upon reasonable request.
