# Supplementary material for: Real-world effectiveness of burosumab vs oral phosphate and active vitamin D in adults with X-linked hypophosphatemia
Source: J Bone Miner Res. 2025 May 2;40(8):973–86. doi: 10.1093/jbmr/zjaf063 (PMC12308825; doi:10.1093/jbmr/zjaf063)
Supplement: Revised_Supplemental_Information_FilledOut_zjaf063 [file revised_supplemental_information_filledout_zjaf063.docx]

**Supplementary Material**

Supplementary Methods

Baseline characteristics used in the propensity score estimation included age, sex, race, weight, height, serum phosphate, 1,25(OH)_2_D, PTH, WOMAC (pain, stiffness, and physical function scores), PROMIS PF, TUG, and medical and treatment history (bowing of the legs, genu valgum, intoeing, osteoarthritis, enthesopathy/bone spurs/osteophytes, spinal cord compression, nontraumatic fracture/pseudofracture, traumatic fracture, number of fractures, spinal surgery, tinnitus, hearing loss, hyperparathyroidism, nephrocalcinosis, hypertension, headache, severe headache, depression, age at diagnosis, history of pediatric Pi/D ever, and any pain or opioid medication at baseline). Ethnicity and country were not included in the propensity score calculation because they are not considered clinical characteristics and do not have an impact on outcomes. Due to 100% of the Pi/D cohort having received Pi/D treatment, “history of Pi/D ever” was not incorporated in the propensity score calculation. Any baseline parameter (eg, demographic, clinical characteristic, medical or treatment history) not included in the propensity score calculation was not expected to show improved balance after implementing inverse probability of treatment weighting.

Supplemental Table 1. Treatment Patterns in the Pi/D Cohort (N=74)

| **Pi/D treatment (N=74)** | **n (%)^a^** | **Discontinued prior to year 1 visit, n (%)** | **Median (IQR) months from discontinuation to year 1 visit** |
| --- | --- | --- | --- |
| Oral phosphate | 61 (82.4) | 20 (32.8) | 6.41 (4.49, 8.15) |
| Calcitriol | 37 (50.0) | 5 (13.5) | 4.80 (3.58, 7.82) |
| Cholecalciferol | 17 (23.0) | 6 (35.3) | 5.41 (4.27, 6.05) |
| Other vitamin D and analogs^b^ | 62 (83.8) | 26 (41.9) | 7.13 (5.56, 8.74) |
| Vitamin D not otherwise specified^b^ | 7 (9.5) | 3 (42.9) | 6.64 (1.61, 8.74) |

IQR, interquartile range; Pi/D, oral phosphate/active vitamin D.

^a^Total N (%) is calculated using the Pi/D cohort sample size of 74.

^b^May include calcitriol and/or cholecalciferol treatments.

**Supplemental Table 2.**  **Biochemistry Measures After** **Inverse Probability of Treatment Weighting^a,b,c^**

| **Biochemical measure** |  |  | **Baseline** | **Year 1** | **Change** | ***P* value** |
| --- | --- | --- | --- | --- | --- | --- |
| **Serum phosphate concentration, mg/dL** | Burosumab | N | 65.00 | 64.00 | 64.00 | <0.001 |
|  |  | Mean (SE) | 2.11 (0.04) | 2.90 (0.08) | 0.78 (0.08) |  |
|  | Pi/D | N | 32.48 | 30.97 | 30.84 |  |
|  |  | Mean (SE) | 2.18 (0.13) | 2.31 (0.13) | 0.15 (0.14) |  |
| **1,25(OH)_2_D, pg/mL** | Burosumab | N | 56.00 | 54.00 | 47.00 | 0.011 |
|  |  | Mean (SE) | 40.73 (2.24) | 60.06 (3.75) | 19.41 (3.39) |  |
|  | Pi/D | N | 31.36 | 27.09 | 25.84 |  |
|  |  | Mean (SE) | 42.45 (2.81) | 48.92 (3.91) | 5.49 (3.43) |  |
| **PTH, pg/mL** | Burosumab | N | 63.00 | 63.00 | 61.00 | 0.006 |
|  |  | Mean (SE) | 83.40 (6.04) | 69.86 (4.76) | −13.82 (5.00) |  |
|  | Pi/D | N | 32.60 | 31.31 | 31.31 |  |
|  |  | Mean (SE) | 81.84 (13.91) | 93.09 (20.77) | 11.79 (8.10) |  |

1,25(OH)_2_D, 1,25-dihydroxyvitamin D; Pi/D, oral phosphate/active vitamin D; PTH, parathyroid hormone; SE, standard error.

^a^N values are weighted.

^b^Sandwich variance estimator is used to estimate SE.

^c^For inverse probability of treatment weighting, each patient is assigned a weight to make the cohort on average similar to the burosumab cohort, where each patient has a weight of 1.

**Supplemental Table 3. Outcomes in Biochemistry Measures, PROs, and TUG Physical Performance by Cohort Before Inverse Probability of Treatment Weighting**

| **Outcome** |  |  | **Before inverse probability of treatment weighting** | | | |
| --- | --- | --- | --- | --- | --- | --- |
|  |  |  | **Baseline** | **Year 1** | **Change** | ***P* value** |
| **Serum phosphate concentration, mg/dL** | Burosumab | N | 65 | 64 | 64 | <0.001 |
|  |  | Mean (SE) | 2.11 (0.04) | 2.90 (0.08) | 0.78 (0.08) |  |
|  | Pi/D | N | 73 | 72 | 71 |  |
|  |  | Mean (SE) | 2.20 (0.05) | 2.31 (0.05) | 0.14 (0.06) |  |
| **1,25(OH)_2_D, pg/mL** | Burosumab | N | 56 | 54 | 47 | <0.001 |
|  |  | Mean (SE) | 40.73 (2.24) | 60.06 (3.75) | 19.41 (3.39) |  |
|  | Pi/D | N | 72 | 63 | 61 |  |
|  |  | Mean (SE) | 45.79 (2.40) | 45.29 (1.97) | −1.14 (1.89) |  |
| **PTH, pg/mL** | Burosumab | N | 63 | 63 | 61 | 0.066 |
|  |  | Mean (SE) | 83.40 (6.04) | 69.86 (4.76) | −13.82 (5.00) |  |
|  | Pi/D | N | 74 | 73 | 73 |  |
|  |  | Mean (SE) | 88.41 (11.29) | 86.93 (13.39) | −1.39 (4.50) |  |
| **WOMAC pain^a^** | Burosumab | N | 65 | 64 | 64 | 0.002 |
|  |  | Mean (SE) | 38.54 (2.77) | 31.25 (2.91) | −7.50 (2.34) |  |
|  | Pi/D | N | 72 | 73 | 71 |  |
|  |  | Mean (SE) | 28.26 (2.96) | 30.68 (3.05) | 2.25 (1.98) |  |
| **WOMAC stiffness^a^** | Burosumab | N | 65 | 64 | 64 | 0.026 |
|  |  | Mean (SE) | 52.12 (2.92) | 41.80 (2.83) | −10.16 (2.85) |  |
|  | Pi/D | N | 73 | 73 | 72 |  |
|  |  | Mean (SE) | 38.70 (3.50) | 38.70 (3.20) | −0.69 (3.05) |  |
| **WOMAC physical function^a^** | Burosumab | N | 65 | 64 | 64 | 0.078 |
|  |  | Mean (SE) | 34.30 (3.03) | 28.58 (2.90) | −5.68 (1.96) |  |
|  | Pi/D | N | 72 | 72 | 70 |  |
|  |  | Mean (SE) | 30.51 (3.21) | 30.90 (3.05) | −0.23 (2.33) |  |
| **WOMAC total^a^** | Burosumab | N | 65 | 64 | 64 | <0.001 |
|  |  | Mean (SE) | 41.65 (2.68) | 33.88 (2.65) | −7.78 (2.06) |  |
|  | Pi/D | N | 73 | 73 | 72 |  |
|  |  | Mean (SE) | 32.52 (3.25) | 33.44 (3.11) | 0.44 (2.47) |  |
| **PROMIS PF^b^** | Burosumab | N | 65 | 64 | 64 | 0.074 |
|  |  | Mean (SE) | 40.67 (1.10) | 42.19 (1.11) | 1.51 (0.73) |  |
|  | Pi/D | N | 74 | 72 | 72 |  |
|  |  | Mean (SE) | 42.03 (1.07) | 41.92 (1.20) | −0.23 (0.64) |  |
| **TUG, seconds^c^** | Burosumab | N | 55 | 49 | 47 | 0.334 |
|  |  | Mean (SE) | 10.48 (0.83) | 9.50 (0.77) | −1.19 (0.42) |  |
|  | Pi/D | N | 68 | 67 | 65 |  |
|  |  | Mean (SE) | 12.04 (1.08) | 11.93 (0.72) | −0.15 (0.99) |  |

1,25(OH)_2_D, 1,25-dihydroxyvitamin D; IQR, interquartile range, Pi/D, oral phosphate/active vitamin D; PROMIS PF, Patient-Reported Outcomes Measurement Information System Physical Function; PTH, parathyroid hormone; SE, standard error; TUG, Timed Up and Go; WOMAC, Western Ontario and McMaster Universities Osteoarthritis Index.

^a^The WOMAC index is scored on a scale of 0-100. A higher WOMAC score indicates a worse outcome.[35]

^b^PROMIS PF is score of 50 is equivalent to results from an average population.[30]

^c^A higher TUG score indicates an increase in predicted fall risk.[24]

**Supplemental Table 4. PROs and Physical Performance After** **Inverse Probability of Treatment Weighting^a,b,c^**

| **Outcome** |  |  | **Baseline** | **Year 1** | **Change** | ***P* value** |
| --- | --- | --- | --- | --- | --- | --- |
| **WOMAC pain^d^** | Burosumab | N | 65.00 | 64.00 | 64.00 | 0.004 |
|  |  | Mean (SE) | 38.54 (2.77) | 31.25 (2.91) | −7.50 (2.34) |  |
|  | Pi/D | N | 31.31 | 32.47 | 31.17 |  |
|  |  | Mean (SE) | 34.23 (5.32) | 39.17 (5.71) | 4.47 (3.23) |  |
| **WOMAC stiffness^d^** | Burosumab | N | 65.00 | 64.00 | 64.00 | 0.086 |
|  |  | Mean (SE) | 52.12 (2.92) | 41.80 (2.83) | −10.16 (2.85) |  |
|  | Pi/D | N | 31.31 | 32.47 | 31.18 |  |
|  |  | Mean (SE) | 48.16 (5.51) | 47.60 (5.66) | −1.79 (3.68) |  |
| **WOMAC physical function^d^** | Burosumab | N | 65.00 | 64.00 | 64.00 | 0.006 |
|  |  | Mean (SE) | 34.30 (3.03) | 28.58 (2.90) | −5.68 (1.96) |  |
|  | Pi/D | N | 31.31 | 32.47 | 31.17 |  |
|  |  | Mean (SE) | 27.55 (4.80) | 34.85 (5.45) | 6.77 (4.85) |  |
| **WOMAC total^d^** | Burosumab | N | 65.00 | 64.00 | 64.00 | 0.005 |
|  |  | Mean (SE) | 41.65 (2.68) | 33.88 (2.65) | −7.78 (2.06) |  |
|  | Pi/D | N | 31.31 | 32.47 | 31.18 |  |
|  |  | Mean (SE) | 36.65 (4.62) | 40.54 (5.41) | 3.15 (3.37) |  |
| **PROMIS PF^e^** | Burosumab | N | 65.00 | 64.00 | 64.00 | 0.018 |
|  |  | Mean (SE) | 40.67 (1.10) | 42.19 (1.11) | 1.51 (0.73) |  |
|  | Pi/D | N | 32.60 | 30.00 | 30.00 |  |
|  |  | Mean (SE) | 42.52 (2.18) | 41.42 (2.15) | −1.64 (1.11) |  |
| **TUG, seconds^f^** | Burosumab | N | 55.00 | 49.00 | 47.00 | 0.011 |
|  |  | Mean (SE) | 10.48 (0.83) | 9.50 (0.77) | −1.19 (0.42) |  |
|  | Pi/D | N | 24.08 | 24.92 | 22.43 |  |
|  |  | Mean (SE) | 9.87 (0.45) | 10.59 (0.70) | 0.55 (0.43) |  |

Pi/D, oral phosphate/active vitamin D; PRO, patient-reported outcome; PROMIS PF, Patient-Reported Outcomes Measurement Information System Physical Function; SE, standard error; TUG, Timed Up and Go; WOMAC, Western Ontario and McMaster Universities Osteoarthritis Index.

^a^N values are weighted.

^b^Sandwich variance estimator is used to estimate SE.

^c^For inverse probability of treatment weighting, each patient is assigned a weight to make the cohort on average similar to the burosumab cohort, where each patient has a weight of 1.

^d^The WOMAC index is scored on a scale of 0-100. A higher WOMAC score indicates a worse outcome.[35]

^e^PROMIS PF is score of 50 is equivalent to results from an average population.[30]

^f^A higher TUG score indicates more impairment in mobility and physical ability.[24]

**Supplemental Table 5. Baseline Demographics, Clinical Characteristics, and Medical and Treatment History of the Burosumab Cohort by Baseline Pi/D Status**

| **Characteristic** | **Baseline Pi/D status** | | |
| --- | --- | --- | --- |
|  | **Pi/D at baseline**  **(N=28)** | **No Pi/D at baseline**  **(N=37)** | ***P* value** |
| **Age, mean years (SE)** | 40.60 (2.63) | 38.56 (2.03) | 0.536 |
| **Sex, n (%)** |  |  |  |
| Female | 19 (67.9) | 29 (78.4) | 0.339 |
| Male | 9 (32.1) | 8 (21.6) |  |
| **Race, n (%)** |  |  |  |
| White | 16 (57.1) | 33 (89.2) | 0.008 |
| Non-White | 3 (10.7) | 0 |  |
| Unknown/not reported | 9 (32.1) | 4 (10.8) |  |
| **Ethnicity, n (%)** |  |  |  |
| Hispanic or Latino | 3 (10.7) | 3 (8.1) | 0.036 |
| Not Hispanic or Latino | 16 (57.1) | 31 (83.8) |  |
| Other | 9 (32.1) | 3 (8.1) |  |
| **Country, n (%)** |  |  |  |
| Argentina | 0 | 0 | 0.020 |
| Brazil | 1 (3.6) | 2 (5.4) |  |
| Canada | 9 (32.1) | 2 (5.4) |  |
| Chile | 0 | 0 |  |
| Colombia | 1 (3.6) | 0 |  |
| USA | 17 (60.7) | 33 (89.2) |  |
| **Weight, mean kg (SE)** | 72.43 (3.10) | 74.92 (3.59) | 0.616 |
| **Height, mean cm (SE)** | 153.79 (2.08) | 152.95 (1.36) | 0.725 |
| **Body mass index, mean (SE)** | 30.07 (1.10) | 32.20 (1.65) | 0.289 |
| **Serum phosphate concentration (mg/dL), mean (SE)** | 2.08 (0.08) | 2.14 (0.05) | 0.434 |
| **1,25(OH)_2_D (pg/mL), mean (SE)** | 44.77 (3.27) | 37.71 (2.99) | 0.120 |
| **PTH (pg/mL), mean (SE)** | 87.78 (11.85) | 79.91 (5.44) | 0.550 |
| **WOMAC score, mean (SE)^a^** |  |  |  |
| Pain | 37.32 (4.48) | 39.46 (3.54) | 0.706 |
| Physical function | 32.83 (5.05) | 35.41 (3.75) | 0.676 |
| Stiffness score | 50.00 (4.64) | 53.72 (3.78) | 0.533 |
| Total | 40.05 (4.87) | 42.86 (3.88) | 0.429 |
| **PROMIS PF, mean (SE)^b^** | 40.45 (1.55) | 40.84 (1.55) | 0.862 |
| **TUG, mean (SE)^c^** | 11.72 (1.69) | 9.37 (0.41) | 0.187 |
| **Bowing of legs, n (%)** | 23 (82.1) | 30 (81.1) | 0.913 |
| **Genu valgum, n (%)** | 6 (21.4) | 4 (10.8) | 0.240 |
| **Intoeing, n (%)** | 15 (53.6) | 18 (48.7) | 0.694 |
| **Osteoarthritis, n (%)** | 14 (50.0) | 16 (43.2) | 0.588 |
| **Enthesopathy/bone spurs/osteophytes, n (%)** | 16 (57.1) | 20 (54.1) | 0.804 |
| **Spinal cord compression, n (%)** | 4 (14.3) | 7 (18.9) | 0.622 |
| **Nontraumatic fracture/pseudofracture, n (%)** | 11 (39.3) | 11 (29.7) | 0.420 |
| **Traumatic fracture, n (%)** | 8 (28.6) | 8 (21.6) | 0.520 |
| **Number of fractures ever, mean (SE)^d^** | 7.86 (4.56) | 3.14 (0.77) | 0.326 |
| **Spinal surgery, n (%)** | 2 (7.1) | 3 (8.1) | 0.885 |
| **Tinnitus, n (%)** | 8 (28.6) | 12 (32.4) | 0.738 |
| **Hearing loss, n (%)** | 8 (28.6) | 10 (27.0) | 0.890 |
| **Hyperparathyroidism, n (%)** | 7 (25.0) | 8 (21.6) | 0.749 |
| **Nephrocalcinosis, n (%)** | 4 (14.3) | 5 (13.5) | 0.929 |
| **Hypertension, n (%)** | 8 (28.6) | 6 (16.2) | 0.230 |
| **Headache, n (%)** | 9 (32.1) | 10 (27.0) | 0.653 |
| **Severe headache, n (%)** | 6 (21.4) | 9 (24.3) | 0.784 |
| **Age at XLH diagnosis, mean years (SE)** | 8.81 (3.45) | 11.42 (2.77) | 0.553 |
| **History of Pi/D ever, n (%)** | 27 (96.4) | 30 (81.1) | 0.062 |
| **History of pediatric Pi/D, n (%)** | 24 (85.7) | 27 (73.0) | 0.216 |
| **History of adult Pi/D, n (%)** | 26 (92.9) | 16 (43.2) | <0.001 |
| **Pi/D at baseline, n (%)** | 28 (100.0) | 0 | <0.001 |
| **Any pain medication at baseline, n (%)** | 16 (57.1) | 23 (62.2) | 0.683 |
| **Any opioid medication at baseline, n (%)** | 4 (14.3) | 4 (10.8) | 0.673 |

1,25(OH)_2_D, 1,25-dihydroxyvitamin D; Pi/D, oral phosphate/active vitamin D; PROMIS PF, Patient-Reported Outcomes Measurement Information System Physical Function; PTH, parathyroid hormone; SE, standard error; TUG, Timed Up and Go; WOMAC, Western Ontario and McMaster Osteoarthritis Index; XLH, X-linked hypophosphatemia.

^a^The WOMAC index is scored on a scale of 0-100. A higher WOMAC score indicates a worse outcome.[35]

^b^PROMIS PF is score of 50 is equivalent to results from an average population.[30]

^c^A higher TUG score indicates an increase in predicted fall risk.[24]

^d^Includes traumatic and nontraumatic/pseudofractures.

**Supplemental Table 6. Outcomes in Biochemistry, PROs, and Physical Performance in the Burosumab Cohort by Baseline Pi/D Status**

| **Outcome** | **Baseline Pi/D status** |  | **Year 1** | **Change** | ***P* value** |
| --- | --- | --- | --- | --- | --- |
| **Serum phosphate concentration, mg/dL** | Pi/D at baseline | N | 28 | 28 | 0.336 |
|  |  | Mean (SE) | 2.77 (0.09) | 0.70 (0.12) |  |
|  | No Pi/D at baseline | N | 36 | 36 |  |
|  |  | Mean (SE) | 3.00 (0.12) | 0.85 (0.10) |  |
| **1,25(OH)_2_D, pg/mL** | Pi/D at baseline | N | 22 | 19 | 0.563 |
|  |  | Mean (SE) | 63.63 (6.20) | 22.12 (7.06) |  |
|  | No Pi/D at baseline | N | 32 | 28 |  |
|  |  | Mean (SE) | 57.60 (4.71) | 17.58 (3.15) |  |
| **PTH, pg/mL** | Pi/D at baseline | N | 27 | 27 | 0.270 |
|  |  | Mean (SE) | 68.03 (5.90) | −20.36 (9.06) |  |
|  | No Pi/D at baseline | N | 36 | 34 |  |
|  |  | Mean (SE) | 71.23 (7.13) | −8.62 (5.30) |  |
| **WOMAC pain^a^** | Pi/D at baseline | N | 27 | 27 | 0.569 |
|  |  | Mean (SE) | 28.70 (4.55) | −9.07 (2.85) |  |
|  | No Pi/D at baseline | N | 37 | 37 |  |
|  |  | Mean (SE) | 33.11 (3.81) | −6.35 (3.49) |  |
| **WOMAC stiffness^a^** | Pi/D at baseline | N | 27 | 27 | 0.487 |
|  |  | Mean (SE) | 37.04 (4.50) | −12.50 (4.67) |  |
|  | No Pi/D at baseline | N | 37 | 37 |  |
|  |  | Mean (SE) | 45.27 (3.57) | −8.45 (3.59) |  |
| **WOMAC physical function^a^** | Pi/D at baseline | N | 27 | 27 | 0.591 |
|  |  | Mean (SE) | 25.76 (4.43) | −6.92 (3.14) |  |
|  | No Pi/D at baseline | N | 37 | 37 |  |
|  |  | Mean (SE) | 30.64 (3.86) | −4.77 (2.51) |  |
| **WOMAC total^a^** | Pi/D at baseline | N | 28 | 28 | 0.292 |
|  |  | Mean (SE) | 30.50 (4.45) | −9.50 (3.56) |  |
|  | No Pi/D at baseline | N | 37 | 37 |  |
|  |  | Mean (SE) | 36.34 (3.86) | −6.52 (3.21) |  |
| **PROMIS PF^b^** | Pi/D at baseline | N | 27 | 27 | 0.887 |
|  |  | Mean (SE) | 41.85 (1.46) | 1.39 (1.15) |  |
|  | No Pi/D at baseline | N | 37 | 37 |  |
|  |  | Mean (SE) | 42.44 (1.62) | 1.60 (0.97) |  |
| **TUG, seconds^c^** | Pi/D at baseline | N | 24 | 23 | 0.123 |
|  |  | Mean (SE) | 10.17 (1.47) | −1.84 (0.64) |  |
|  | No Pi/D at baseline | N | 25 | 24 |  |
|  |  | Mean (SE) | 8.86 (0.56) | −0.57 (0.50) |  |

1,25(OH)_2_D, 1,25-dihydroxyvitamin D; Pi/D, oral phosphate/active vitamin D; PRO, patient-reported outcome; PROMIS PF, Patient-Reported Outcomes Measurement Information System Physical Function; PTH, parathyroid hormone; SE, standard error; TUG, Timed Up and Go; WOMAC, Western Ontario and McMaster Osteoarthritis Index.

^a^The WOMAC index is scored on a scale of 0-100. A higher WOMAC score indicates a worse outcome.[35]

^b^PROMIS PF is score of 50 is equivalent to results from an average population.[30]

^c^A higher TUG score indicates an increase in predicted fall risk.[24]

**Supplemental Table 7. Baseline Demographics, Clinical Characteristics, and Medical and Treatment History of Patients in the US and Canada by Cohort**

| **Characteristic** | **Burosumab**  **(N=61)** | **Pi/D**  **(N=23)** | ***P* value** |
| --- | --- | --- | --- |
| **Age, mean years (SE)** | 39.17 (1.69) | 46.98 (3.38) | 0.026 |
| **Sex, n (%)** |  |  |  |
| Female | 45 (73.8) | 20 (87.0) | 0.198 |
| Male | 16 (26.2) | 3 (13.0) |  |
| **Race, n (%)** |  |  |  |
| White | 47 (77.1) | 18 (78.3) | 0.673 |
| Non-White | 2 (3.3) | 0 |  |
| Unknown/not reported | 12 (19.7) | 5 (21.7) |  |
| **Ethnicity, n (%)** |  |  |  |
| Hispanic or Latino | 2 (3.3) | 2 (8.7) | 0.552 |
| Not Hispanic or Latino | 47 (77.1) | 16 (69.6) |  |
| Other | 12 (19.7) | 5 (21.7) |  |
| **Country, n (%)** |  |  |  |
| Canada | 11 (18.0) | 5 (21.7) | 0.700 |
| USA | 50 (82.0) | 18 (78.3) |  |
| **Weight, mean kg (SE)** | 73.96 (2.57) | 74.25 (3.95) | 0.953 |
| **Height, mean cm (SE)** | 153.83 (1.15) | 152.22 (1.84) | 0.464 |
| **Body mass index, mean (SE)** | 31.10 (1.08) | 31.89 (1.92) | 0.712 |
| **Serum phosphate concentration (mg/dL), mean (SE)** | 2.12 (0.05) | 2.28 (0.12) | 0.206 |
| **1,25(OH)_2_D (pg/mL), mean (SE)** | 41.33 (2.39) | 44.92 (4.28) | 0.436 |
| **PTH (pg/mL), mean (SE)** | 83.28 (6.35) | 116.22 (33.02) | 0.337 |
| **WOMAC score, mean (SE)^a^** |  |  |  |
| Pain | 39.02 (2.82) | 24.78 (4.69) | 0.010 |
| Physical function | 34.19 (3.16) | 22.43 (4.94) | 0.053 |
| Stiffness score | 52.46 (2.96) | 37.50 (5.78) | 0.014 |
| Total | 41.89 (3.13) | 28.24 (5.26) | <0.001 |
| **PROMIS PF, mean (SE)^b^** | 40.91 (1.13) | 45.19 (2.07) | 0.059 |
| **TUG, mean (SE)^c^** | 10.50 (0.87) | 10.12 (0.64) | 0.721 |
| **Bowing of legs, n (%)** | 50 (82.0) | 16 (69.6) | 0.217 |
| **Genu valgum, n (%)** | 8 (13.1) | 9 (39.1) | 0.008 |
| **Intoeing, n (%)** | 33 (54.1) | 14 (60.9) | 0.577 |
| **Osteoarthritis, n (%)** | 28 (45.9) | 12 (52.2) | 0.608 |
| **Enthesopathy/bone spurs/osteophytes, n (%)** | 34 (55.7) | 12 (52.2) | 0.770 |
| **Spinal cord compression, n (%)** | 11 (18.0) | 3 (13.0) | 0.584 |
| **Nontraumatic fracture/pseudofracture, n (%)** | 21 (34.4) | 4 (17.4) | 0.128 |
| **Traumatic fracture, n (%)** | 13 (21.3) | 4 (17.4) | 0.690 |
| **Number of fractures ever, mean (SE)^d^** | 5.72 (2.59) | 4.13 (1.55) | 0.601 |
| **Spinal surgery, n (%)** | 5 (8.2) | 0 | 0.157 |
| **Tinnitus, n (%)** | 19 (31.2) | 8 (34.8) | 0.750 |
| **Hearing loss, n (%)** | 17 (27.9) | 8 (34.8) | 0.537 |
| **Hyperparathyroidism, n (%)** | 14 (23.0) | 8 (34.8) | 0.271 |
| **Nephrocalcinosis, n (%)** | 9 (14.8) | 5 (21.7) | 0.444 |
| **Hypertension, n (%)** | 14 (23.0) | 7 (30.4) | 0.480 |
| **Headache, n (%)** | 18 (29.5) | 6 (26.1) | 0.757 |
| **Severe headache, n (%)** | 14 (23.0) | 4 (17.4) | 0.580 |
| **Age at XLH diagnosis, mean years (SE)** | 9.29 (2.12) | 5.39 (2.10) | 0.196 |
| **History of Pi/D ever, n (%)** | 54 (88.5) | 23 (100.0) | 0.090 |
| **History of pediatric Pi/D, n (%)** | 49 (80.3) | 20 (87.0) | 0.479 |
| **History of adult Pi/D, n (%)** | 40 (65.6) | 23 (100.0) | 0.001 |
| **Pi/D at baseline, n (%)** | 26 (42.6) | 23 (100.0) | <0.001 |
| **Any pain medication at baseline, n (%)** | 38 (62.3) | 11 (47.8) | 0.230 |
| **Any opioid medication at baseline, n (%)** | 7 (11.5) | 2 (8.7) | 0.713 |

1,25(OH)_2_D, 1,25-dihydroxyvitamin D; Pi/D, oral phosphate/active vitamin D; PROMIS PF, Patient-Reported Outcomes Measurement Information System Physical Function; PTH, parathyroid hormone; SE, standard error; TUG, Timed Up and Go; WOMAC, Western Ontario and McMaster Osteoarthritis Index; XLH, X-linked hypophosphatemia.

^a^The WOMAC index is scored on a scale of 0-100. A higher WOMAC score indicates a worse outcome.[35]

^b^PROMIS PF is score of 50 is equivalent to results from an average population.[30]

^c^A higher TUG score indicates an increase in predicted fall risk.[24]

^d^Includes traumatic and nontraumatic fractures/pseudofractures.

**Supplemental Table 8. Outcomes in Biochemistry, PROs, and Physical Performance of Cohort Patients in the US and Canada**

| **Outcome** |  |  | **Year 1** | **Change** | ***P* value** |
| --- | --- | --- | --- | --- | --- |
| **Serum phosphate concentration, mg/dL** | Burosumab | N | 60 | 60 | 0.001 |
|  |  | Mean (SE) | 2.88 (0.09) | 0.76 (0.08) |  |
|  | Pi/D | N | 22 | 22 |  |
|  |  | Mean (SE) | 2.44 (0.10) | 0.24 (0.11) |  |
| **1,25(OH)_2_D, pg/mL** | Burosumab | N | 51 | 44 | 0.005 |
|  |  | Mean (SE) | 59.73 (3.94) | 18.38 (3.53) |  |
|  | Pi/D | N | 17 | 17 |  |
|  |  | Mean (SE) | 46.36 (4.40) | −0.12 (4.39) |  |
| **PTH, pg/mL** | Burosumab | N | 59 | 57 | 0.048 |
|  |  | Mean (SE) | 69.29 (4.99) | −14.29 (5.33) |  |
|  | Pi/D | N | 23 | 23 |  |
|  |  | Mean (SE) | 123.20 (39.59) | 6.98 (10.21) |  |
| **WOMAC pain^a^** | Burosumab | N | 60 | 60 | 0.001 |
|  |  | Mean (SE) | 29.42 (2.91) | −9.83 (1.86) |  |
|  | Pi/D | N | 23 | 23 |  |
|  |  | Mean (SE) | 26.52 (5.31) | 1.74 (2.15) |  |
| **WOMAC stiffness^a^** | Burosumab | N | 60 | 60 | 0.051 |
|  |  | Mean (SE) | 40.63 (2.92) | −11.67 (2.83) |  |
|  | Pi/D | N | 23 | 23 |  |
|  |  | Mean (SE) | 35.87 (5.46) | −1.63 (3.45) |  |
| **WOMAC physical function^a^** | Burosumab | N | 60 | 60 | 0.035 |
|  |  | Mean (SE) | 26.99 (2.93) | −7.16 (1.87) |  |
|  | Pi/D | N | 23 | 23 |  |
|  |  | Mean (SE) | 22.83 (5.09) | 0.40 (2.94) |  |
| **WOMAC total^a^** | Burosumab | N | 60 | 60 | <0.001 |
|  |  | Mean (SE) | 32.34 (3.00) | −9.55 (2.23) |  |
|  | Pi/D | N | 23 | 23 |  |
|  |  | Mean (SE) | 28.41 (5.34) | 0.17 (2.87) |  |
| **PROMIS PF^b^** | Burosumab | N | 60 | 60 | 0.100 |
|  |  | Mean (SE) | 42.67 (1.14) | 1.74 (0.76) |  |
|  | Pi/D | N | 22 | 22 |  |
|  |  | Mean (SE) | 44.89 (2.42) | −0.72 (1.31) |  |
| **TUG, seconds^c^** | Burosumab | N | 47 | 46 | 0.002 |
|  |  | Mean (SE) | 9.45 (0.80) | −1.22 (0.42) |  |
|  | Pi/D | N | 20 | 20 |  |
|  |  | Mean (SE) | 10.71 (0.78) | 0.51 (0.32) |  |

1,25(OH)_2_D, 1,25-dihydroxyvitamin D; Pi/D, oral phosphate/active vitamin D; PRO, patient-reported outcome; PROMIS PF, Patient-Reported Outcomes Measurement Information System Physical Function; PTH, parathyroid hormone; SE, standard error; TUG, Timed Up and Go; WOMAC, Western Ontario and McMaster Osteoarthritis Index.

^a^The WOMAC index is scored on a scale of 0-100. A higher WOMAC score indicates a worse outcome.[35]

^b^PROMIS PF is score of 50 is equivalent to results from an average population.[30]

^c^A higher TUG score indicates an increase in predicted fall risk.[24]

**Supplemental Figure 1. Proportion of Patients Achieving Improvement > MCID WOMAC Scores at Year 1 Visit Before Inverse Probability of Treatment Weighting**


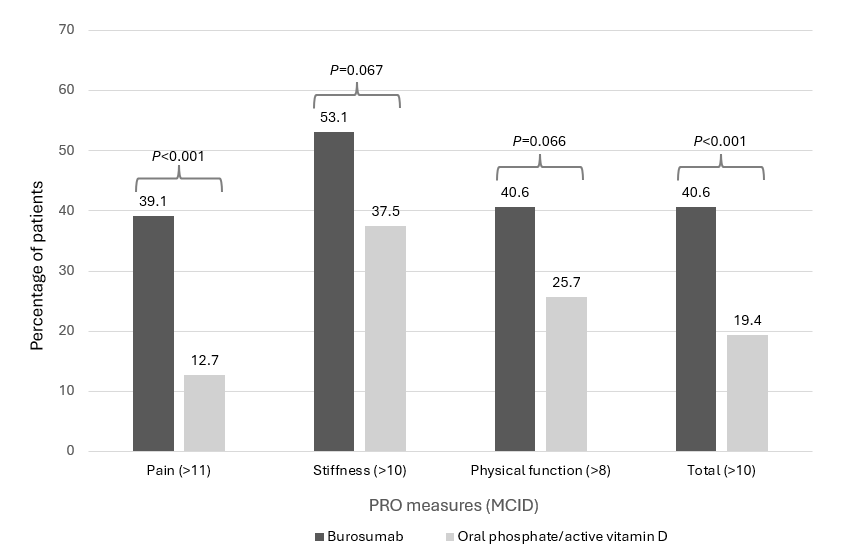


MCID, minimal clinically important difference; PRO, patient-reported outcome; WOMAC, Western Ontario and McMaster Osteoarthritis.

| Country | Name (Last) | Name (First) | Affiliation |
| --- | --- | --- | --- |
| Argentina | Arcari | Andrea | Hospital de Niños Ricardo Gutierrez |
| Argentina | Brunetto | Oscar | Hospital Pedro de Elizalde |
| Argentina | Douthat | Walter Guillermo | Hospital Privado Universitario de Córdoba |
| Brazil | Matsunaga Martin | Regina | Division of Endocrinology and Metabolism  Hospital das Clinicas da Universidade de São Paulo (HCFMUSP) |
| Brazil | Moreira | Carolina A. | Internal Medicine and Endocrine Division (SEMPR) of Federal University of Parana, Academic Research Center of Pro-Renal Institute |
| Canada | Basak | Sanjukta | British Columbia Children's Hospital |
| Canada | Glorieux | Francis | Shriners Hospitals for Children |
| Canada | Khan | Sarah | Bone Research and Education Clinic and Credit Valley Hospital |
| Canada | Ward | Leanne | Children’s Hospital of Eastern Ontario Research Institute |
| Chile | Florenzano | Pablo | Pontificia Universidad Católica de Chile |
| Colombia | Baquero Rodriguez | Richard | Hospital Universitario San Vicente Fundación, Universidad de Antioquia |
| Colombia | Meza-Martinez | Adriana | Hospital Infantil Universitario de San José |
| US | Ashraf | Ambika P. | University of Alabama at Birmingham |
| US | Bowden | Sasigarn | Nationwide Children's Hospital |
| US | Bradley P. | Dixon | University of Colorado School of Medicine |
| US | Carpenter | Thomas | Yale University |
| US | Crane | Janet | The Johns Hopkins University School of Medicine |
| US | Dahir | Kathryn M. | Vanderbilt University Medical Center |
| US | Glass | Ian | Seattle Children's Hospital |
| US | Gottesman | Gary | Washington University School of Medicine |
| US | Gyuricsko | Eric | Eastern Virginia Medical School at Macon & Joan Brock Virginia Health Sciences Old Dominion University |
| US | Holm | Ingrid | Boston Children’s Hospital |
| US | Imel | Erik | Indiana University School of Medicine |
| US | Ing | Steven | The Ohio State University Wexner Medical Center |
| US | Jan de Beur | Suzanne | University of Virginia |
| US | Levine | Michael | Children's Hospital of Philadelphia |
| US | Paloian | Neil | University of Wisconsin School of Medicine & Public Health |
| US | Portale | Anthony | University of California San Francisco Medical Center |
| US | Rodriguez-Buritica | David | The University of Texas Health Science Center at Houston |
| US | Ryabets-Lienhard | Anna | Children’s Hospital of Los Angeles, University of Southern California, Keck School of Medicine |
| US | Simmons | Jill | Vanderbilt University Medical Center |
| US | Singh | Puja | University of California San Diego, Rady Children's Hospital San Diego |
| US | Tabatabai | Laila | Houston Methodist Research Institute |
| US | Wasserman | Halley | Cincinnati Children's Hospital |
| US | Weber | Thomas | Duke University Medical Center |

**Supplemental Appendix:** **DMP Investigator Group Information**
